# The role of m6A RNA methylation regulator in meningioma

**DOI:** 10.18632/aging.205163

**Published:** 2023-10-31

**Authors:** Yu Yang, Liqin Luo, Zhiwu Zhou

**Affiliations:** 1 Department of Neurosurgery, Jiangxi Provincial People’s Hospital, Nanchang 330006, Jiangxi, China; 2 The First Affiliated Hospital of Nanchang Medical College, Nanchang 330006, Jiangxi, China; 3 Nanchang First Retired Cadre Rest House of Jiangxi Military Region, Nanchang 330006, Jiangxi, China; 4 Department of Gastrointestinal Surgery, Jiangxi Provincial People’s Hospital, Nanchang 330006, Jiangxi, China

**Keywords:** m6A methylation, meningiomas, risk score, overall survival (OS)

## Abstract

Meningiomas are common intracranial tumors, and the effect of surgical resection is often unsatisfactory. N6-Methyladenosine (m6A)-related regulator expression levels are related to cancer occurrence and development. This study aimed to investigate the roles of m6A RNA methylation regulators in meningiomas, as these are currently unclear. Two m6A methylation-regulated genes (METTL3 and IGF2BP2) were identified as survival-associated linear models for RiskScore through bioinformatics analysis. Univariate and multivariate Cox regression analyses showed that the overall survival of patients with meningioma in the high-risk group was substantially shorter than that in the low-risk group. Weighted gene co-expression network analysis constructed a co-expression network based on the m6A methylation model (RiskScore). Gene Ontology and the Kyoto Encyclopedia of Genes and Genomes analyses identified the biological processes of hub module gene behavior, and Cytoscape constructed an m6A methylation-related gene regulatory network. *In vitro* experiments verified that the mRNA and protein expression levels of METTL3 and IGF2BP2 were lower in meningioma cells than in normal meningioma cells. Therefore, central regulators of m6A methylation (METTL3 and IGF2BP2) could potentially serve as novel therapeutic targets in meningioma. Subsequently, a novel methylation signature (RiskScore) was developed for prognostic prediction in patients with meningioma.

## INTRODUCTION

Meningiomas originate from benign intracranial tumors of arachnoid cells. Despite the surgical removal of most meningiomas, some patients experience tumor recurrence or clinical disease progression, leading to increased morbidity and mortality [1–3]. Therefore, finding effective diagnostic markers, prognostic indicators, and treatment targets is of great significance for better diagnosis and treatment of meningiomas.

N6-Methyladenosine (m6A), is a modification occurring at the N6 position of RNA adenine (A), which is the most common and abundant internal modification on eukaryotic RNA molecules [4]. Many studies have confirmed that m6A methylation regulatory genes change the expression of target genes by modifying them, thereby regulating cell self-renewal and differentiation, and participation in the pathogenesis and progression of cancer [5, 6]. IGF2BP1 recognizes the m6A site of paternally expressed gene 10 (PEG10) mRNA, bind to the 3' untranslated region and polyadenylic acid binding protein 1, and PEG10 mRNA. This subsequently promotes PEG10 protein expression, thereby accelerating the endometrial cancer cycle [7]. In glioblastoma, [8] METTL3 exerts oncogenic effects by regulating the mRNA expression of splicing factors and alternative splicing isoforms. In epithelial ovarian cancer, [9] ALKBH5 inhibits autophagy and promotes cell proliferation and invasion. However, the relationship between regulators of m6A RNA modification and meningiomas remain unknown and warrants further investigation.

In this study, meningioma sample datasets were obtained from the Gene Expression Omnibus (GEO; http://www.ncbi.nlm.nih.gov/geo/) database, and m6A RNA methylation regulators were used to analyze the expression profiles and prognostic value. The characteristics of prognostic hub genes and methylation regulatory genes were determined through univariate and multivariate Cox analyses and least absolute shrinkage and selection operator (LASSO) Cox regression analysis. Weighted gene co-expression network analysis (WGCNA) was used to construct a co-expression network of clinical features with RiskScore, and enrichment analysis was used to determine the regulation of m6A RNA methylation-related genes in the pathogenesis of meningiomas. Finally, an m6A methylation-regulation network was constructed. In short, the results of this study may provide a powerful reference for individualized treatment of meningiomas.

## RESULTS

### The expression level of m6A RNA methylation regulator in meningioma and normal tissues

**Figure 1:**
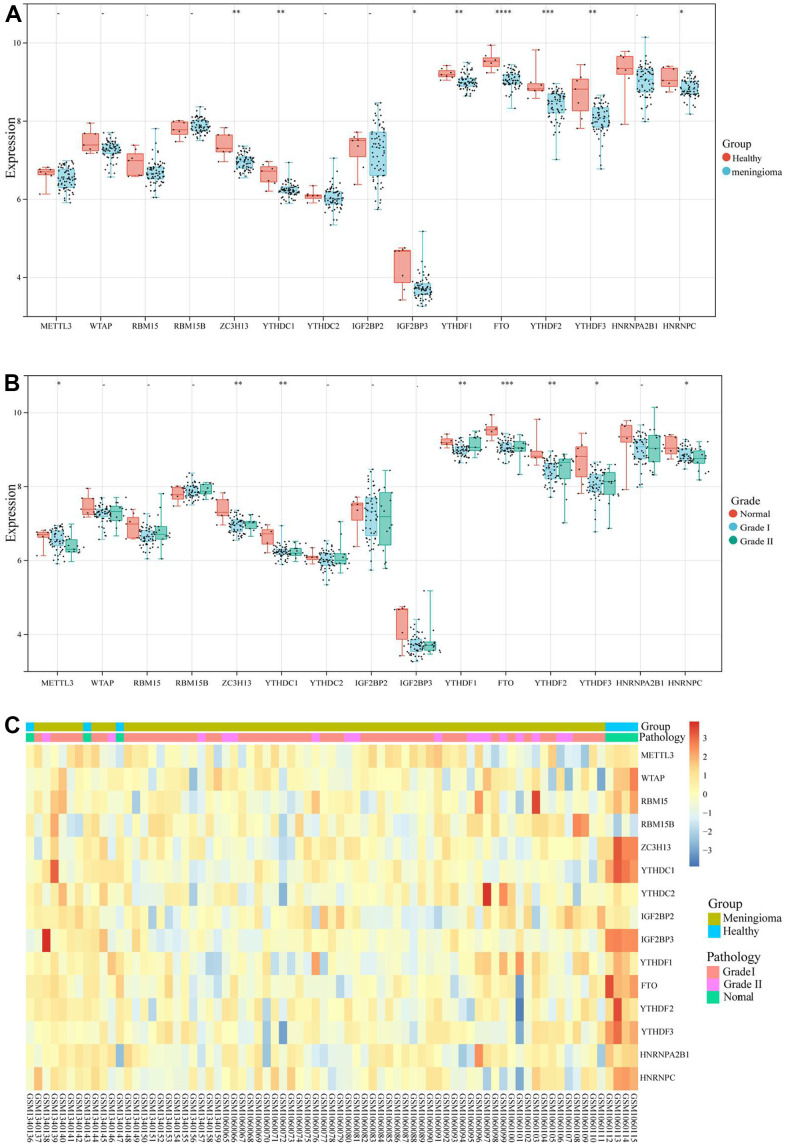
**m6A regulatory genes expression level of GSE55609, GSE43290 datasets.** (**A**) The expression levels of 15 m6A regulatory genes in healthy samples and meningioma samples. (**B**) The expression levels of 15 m6A regulatory genes in different WHO grades (Grade I, Grade II) meningioma samples and normal samples. (**C**) Heatmap of 15 m6A regulatory genes expression level. *<0.05, **<0.01, ***<0.001.

The datasets (GSE55609 and GSE43290) were merged and processed in batches, and the 20 m6A regulatory genes were matched separately. Fifteen m6A regulatory genes were differentially expressed in meningioma and normal samples. Interestingly, the expression levels of ZC3H13, Y1HDC1, IGFBP3, YIHDF1, FTO, YTHDF2, YTHDF3, and HNRNPC in normal samples were substantially higher than those in tumor tissues (Figure 1A); the expression levels of the samples were also notably different between meningioma (Grade I and II) and normal tissues (Figure 1B, 1C). ZC3H13 and YTHDF3 were moderately correlated (COR = 0.59), and there were two negatively correlated genes (RAM15B and METTL3). Therefore, it was speculated that dysregulation of the m6A regulatory gene played a crucial role in the mechanism of meningiomas.

### Using m6A RNA methylation regulator as a prognostic factor for meningiomas

To further verify the clinical effects of m6A methylation, a prognostic analysis of 18 m6A regulatory genes was conducted in the GSE16581 dataset. First, univariate and multivariate Cox regressions screened for prognosis-related and independent prognostic m6A regulators (Table 1). We believed that IGFBP2 and METTL3 were survival-related m6A regulatory genes with critical prognostic value in meningiomas.

**Table 1:** Univariate and multivariate Cox regression analysis results of m6A RNA methylation regulator.

	**Univariant Cox analysis**		
**Characteristics**	**Hazard.Ratio**	**CI95**	***P.*Value**
METTL3	0.11	0.01-1.15	0.066*
METTL14	1.12	0.07-16.97	0.933
KIAA1429	2.01	0.32-12.56	0.453
WTAP	0.88	0.32-2.4	0.801
RBM15	3.21	0.17-61.93	0.44
RBM15B	0.83	0.07-10.54	0.886
ZC3H13	1.36	0.15-12.3	0.785
YTHDC1	1.21	0.28-5.14	0.796
YTHDC2	0.81	0.2-3.22	0.761
IGF2BP2	0.57	0.26-1.23	0.152*
IGF2BP3	0.93	0.06-15.55	0.961
YTHDF1	2.83	0.22-35.82	0.422
ALKBH5	4.83	0.38-61.72	0.226
FTO	5.61	0.43-73.08	0.188*
YTHDF2	0.36	0.05-2.42	0.294
YTHDF3	2.21	0.25-19.71	0.476
HNRNPA2B1	0.79	0.08-8.14	0.84
HNRNPC	0.59	0.27-1.27	0.175*
	**Multivariant Cox analysis**		
**Characteristics**	**Hazard.Ratio**	**CI95**	***P*.Value**
METTL3	0.05	0-0.74	0.03*
IGF2BP2	0.39	0.16-0.98	0.045*
FTO	2.42	0.14-42.7	0.547
HNRNPC	0.5	0.18-1.44	0.2

**Figure 2:**
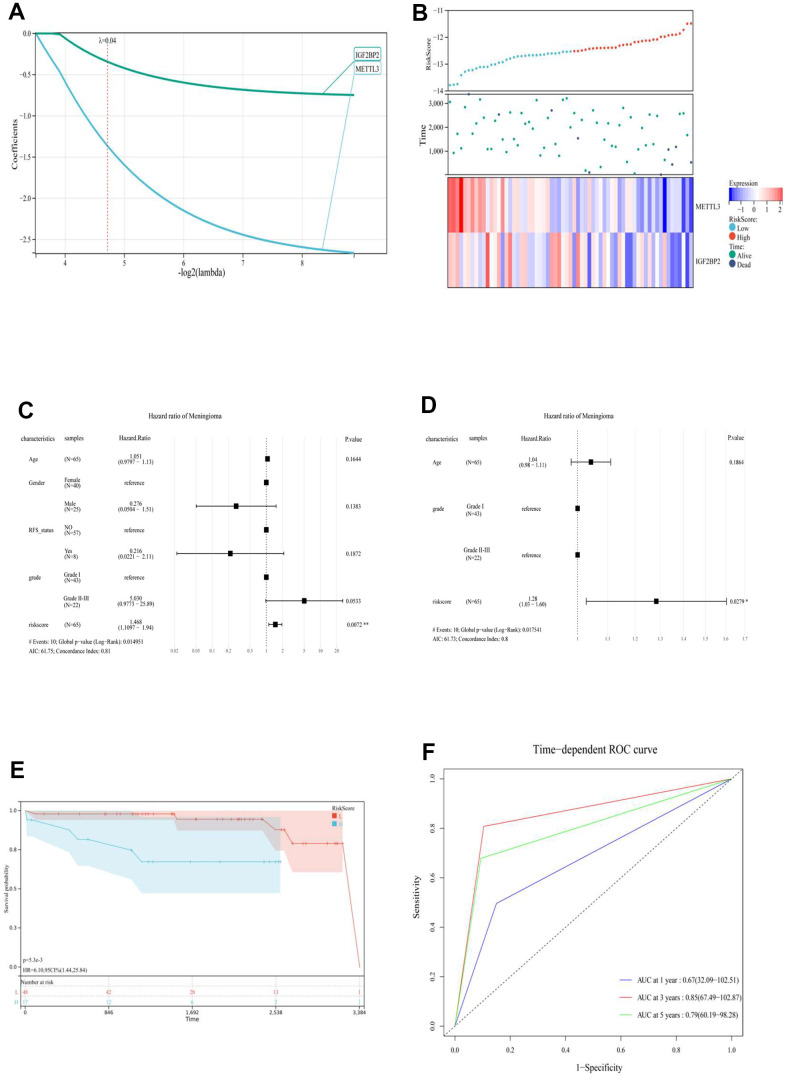
(**A**) LASSO Regression was applied to constructed prognostic prediction model of the m6A regulator. (**B**) RiskScore, survival time and status of each patient (GSE16581), and the expression of two m6A regulatory genes (IGFBP2 and METTL3). (top) RiskScore of each meningioma patient. Blue represents low risk and red represents high risk. (Middle) The survival time and status of each patient with meningioma. (Bottom) The heat map showed the expression of two m6A regulatory genes (IGFBP2 and METTL3) in each patient with meningioma. Blue to red indicated the expression of m6A regulatory genes from low to high expression. Univariate (**C**) and multivariate (**D**) Cox regression analysis to develop the risk scores in meningioma. (**E**) Kaplan-Meier curve analysis of high-risk group and low risk group. (**F**) ROC curve showing the supporting the performance of prognostic prediction model. ROC, receiver operating characteristic; L, low risk score group. H, high risk score group.

**Figure 3:**
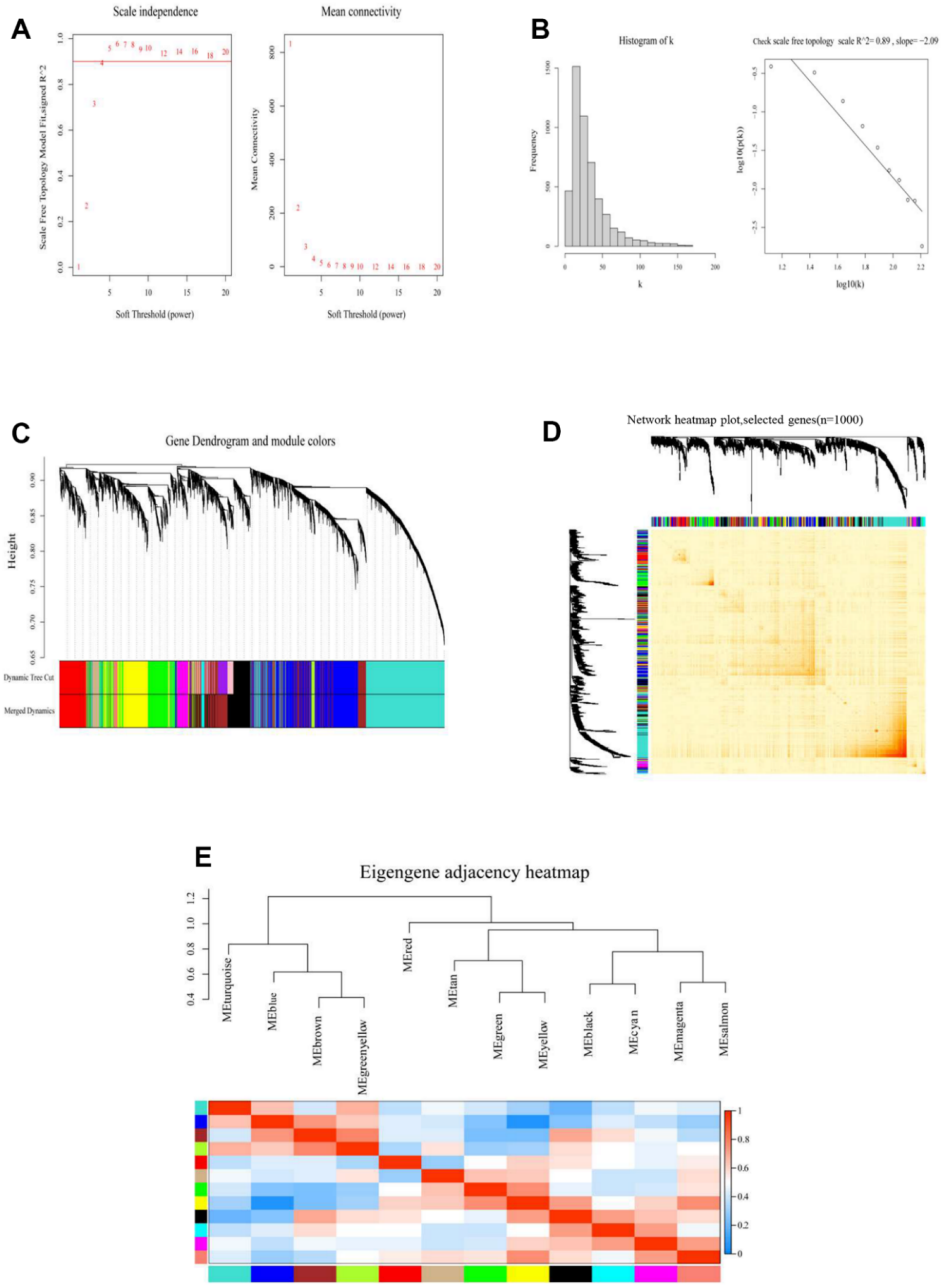
**Constructed a RiskScore-based gene co-expression network through WGCNA.** (**A**) Analyzed the scale-free fitting index of various soft threshold powers (β) (left). Analyzed the average connectivity of various soft threshold powers (right). (**B**) When β = 4, checked the scale-free topology. (**C**) According to cutheight = 0.2, the minimum number of genes was 30, the visualization of Gene Dendrogram and module colors, the dendrograms of all genes were clustered based on a dissimilarity measure (1-TOM). (**D**) Network heatmap plot (selected m6A genes, n=1000). (**E**) Eigengene adjacency heatmap of different modules.

Second, a risk-scoring system based on the level of gene expression and risk coefficient was constructed to comprehensively explore the relationship between the expression level of the m6A regulator and prognosis of meningioma. To avoid overfitting the model construction, LASSO regression was used, and a risk model for the two prognostic genes was constructed based on partial likelihood deviation, lambda value (lambda = 0.04) (Figure 2A, 2B), and RiskScore = (–0.34 * IGF2BP2) + (–1.36 * METTL3). Moreover, the “cutoff” package was used to identify the best cutoff value to group the prognostic models (high-risk and low-risk groups). Overall, univariate (C-index: 0.81) and multivariate Cox (C-index: 0.8) regression analyses further confirmed that the RiskScore was an independent prognostic factor for the prognosis of meningioma (Figure 2C, 2D). The high-risk group had a shorter survival time than the low-risk group (*p* < 5.3e-3) (Figure 3E). The receiver operating characteristic (ROC) curve was used to evaluate the prognostic model and prediction accuracy of the two m6A genes (Figure 2F). The prognostic RiskScore models showed 1-, 3-, and 5-year area under the ROC curve (AUC; 0.67, 0.85, and 0.79, respectively). RiskScore proved to be an independent prognostic factor of meningiomas with good sensitivity and specificity.

### Construction of m6A RNA methylation gene network based on the WGCNA

**Figure 4:**
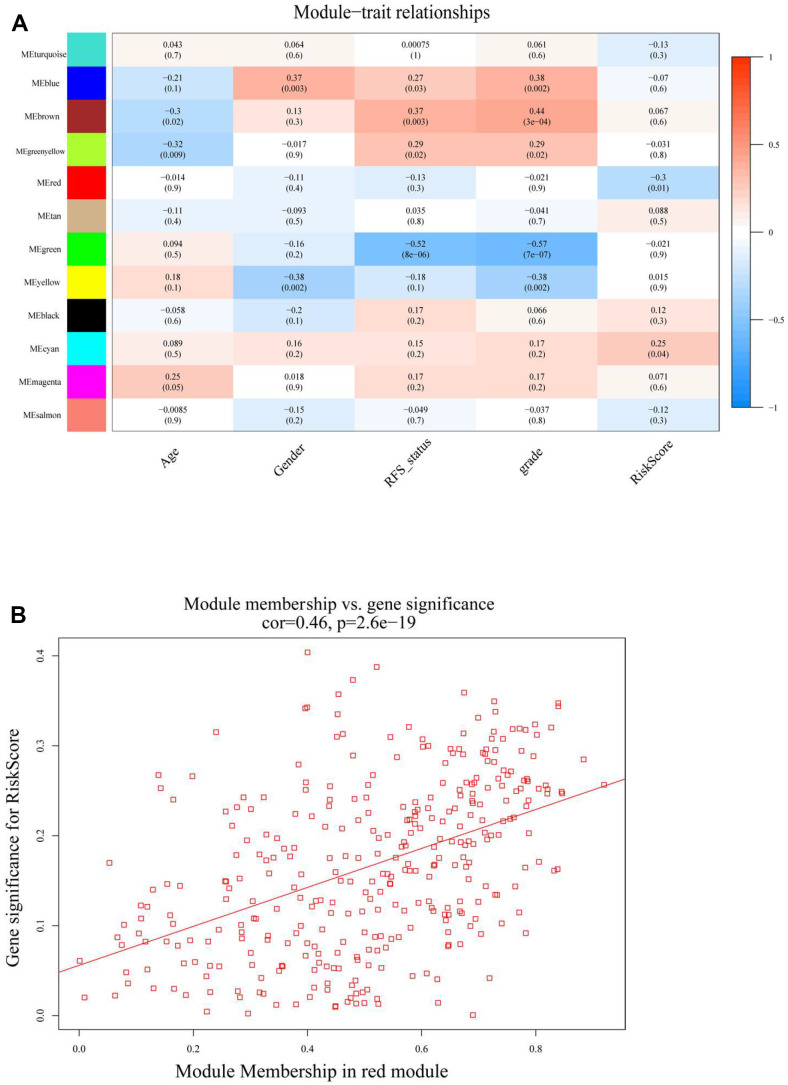
(**A**) Heatmap of the correlation between module (MEs) and trait characteristics (included RiskScore) of meningioma. (**B**) The correlation analysis between module membership (MM) in red module and gene significance (GS) for RiskScore.

The results revealed that RiskScore was an independent prognostic factor for meningiomas. The gene regulation of methylation in meningiomas was clarified by a designed gene regulatory network associated with the RiskScore; this was also used to establish separate groups (high- and low-risk groups) through the “cutoff” package, and WGCNA was performed. The soft threshold power was set to four (R^2^ = 0.89) and modules were merged (threshold value = 0.2) to obtain 12 modules (Figure 3A–3D). The network heatmap indicated close relation between the modules (Figure 3D), and the eigengene adjacency heatmap showed that the modules were independent of each other (Figure 3E). The module-trait relationships heatmap (Figure 4A) helped in determining that the RiskScore was negatively correlated with the red module (COR = –0.3, *p* = 0.01).

**Figure 5:**
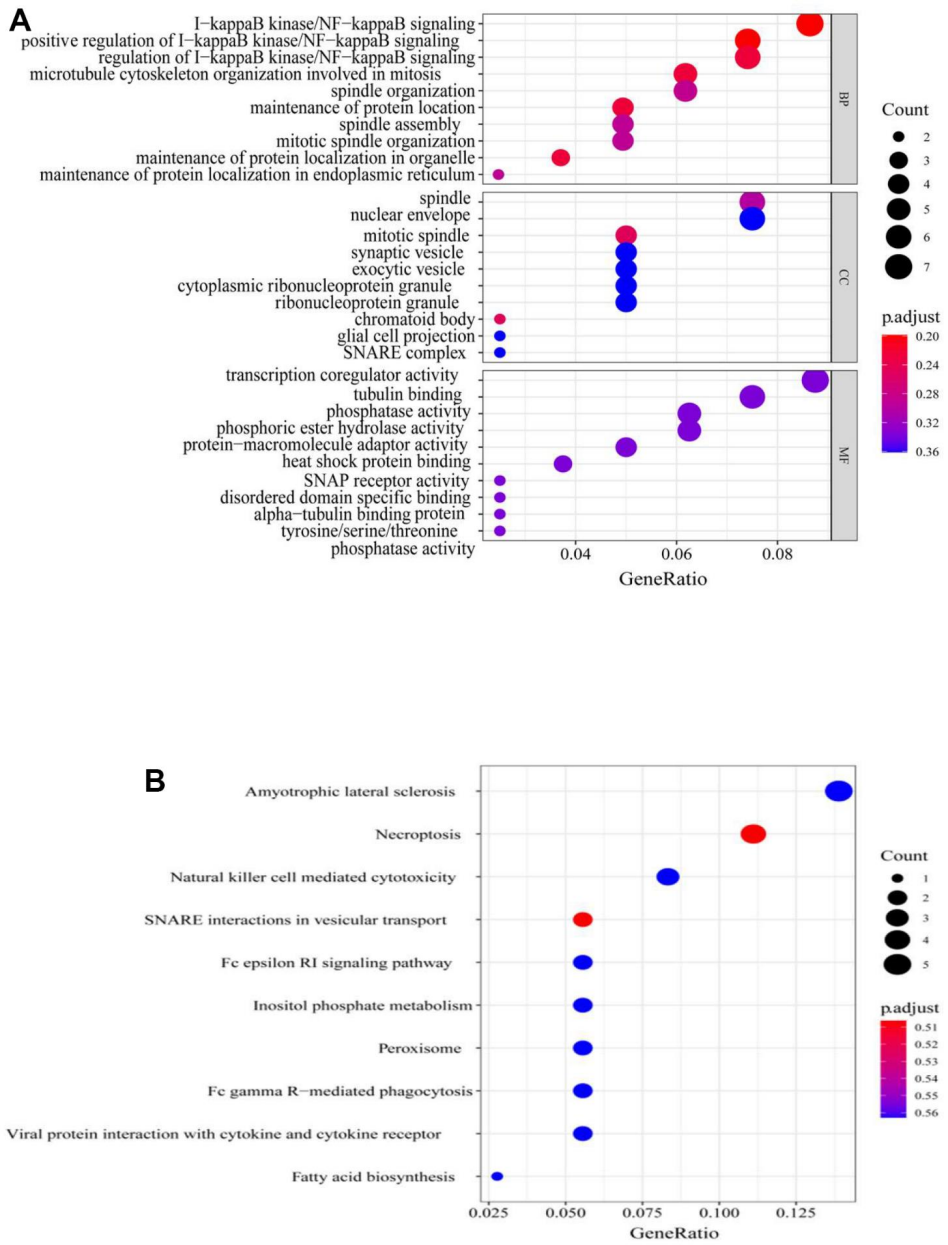
**GO and KEGG analysis of genes in red module.** (**A**) GO analysis, (**B**) KEGG analysis. GO, Gene Ontology; KEGG, Kyoto Encyclopedia of Genes and Genomes.

To further understand the features of the characteristic genes in the red module, the correlation between module membership and significance of the gene was first analyzed (COR = 0.46, *p* = 2.6e-19) (Figure 4B and Supplementary Table 2); this accelerated the understanding of the regulatory mechanism of m6A methylation. Next, Gene Ontology (GO) function and Kyoto Encyclopedia of Genes and Genomes (KEGG) pathway analyses were performed for all genes in the red module (Figure 5A, 5B). The red module GO (Biological Progress) analysis mainly comprised I-kappaB kinase/NF-kappaB signaling, and KEGG pathway analysis comprised necroptosis and natural killer cell-mediated cytotoxicity. The RiskScore regulatory network was identified, and the disease gene characteristics and potential biological mediation pathways of RiskScore as independent prognostic factors were preliminarily described.

### m6A RNA methylation regulator gene network visualization

**Figure 6:**
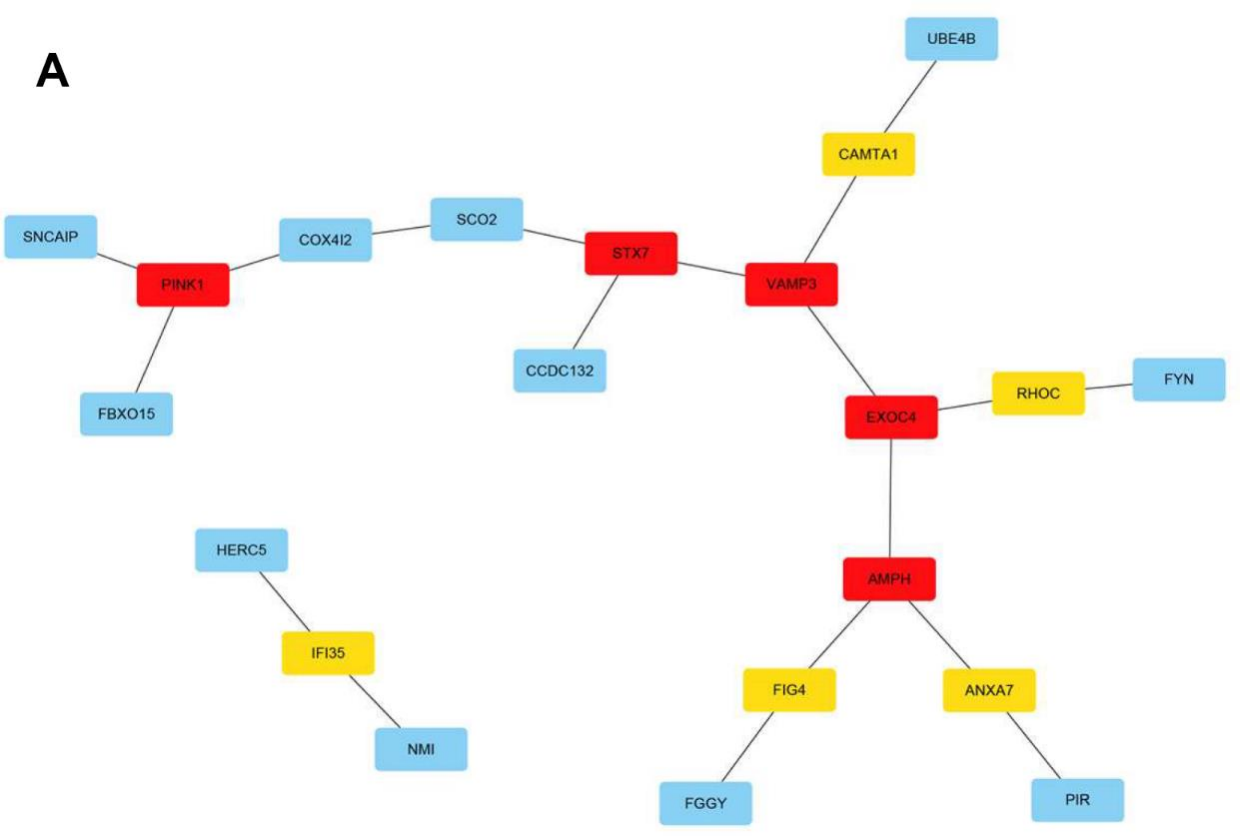
**The m6A RNA methylation regulation gene network in meningiomas.** (**A**) Visualized by Cytoscape, cytoHubba screened important gene modules. The darker the color of the hub node, the higher the score, and the more inclined to key genes.

The Search Tool for the Retrieval of Interacting Genes/Proteins (STRING; https://cn.string-db.org/) database serves as an online platform that evaluates and integrates protein-protein interactions and transmits mutual information between organisms [10]. Here, all genes in the red module (n = 100) were analyzed. Moreover, Cytoscape software was used for network visualization and processing (Figure 6A). The m6A RNA methylation regulatory network landscape in meningiomas was also described. In other words, the mechanism of meningiomas could involve a gene network regulated by m6A RNA methylation and occur due to changes in the abovementioned genes. The results of this study lay a foundation for future research on the genetic landscape of meningiomas.

### Low expression of m6A methylated regulators (METTL3 and IGF2BP2) in meningioma cells

**Figure 7:**
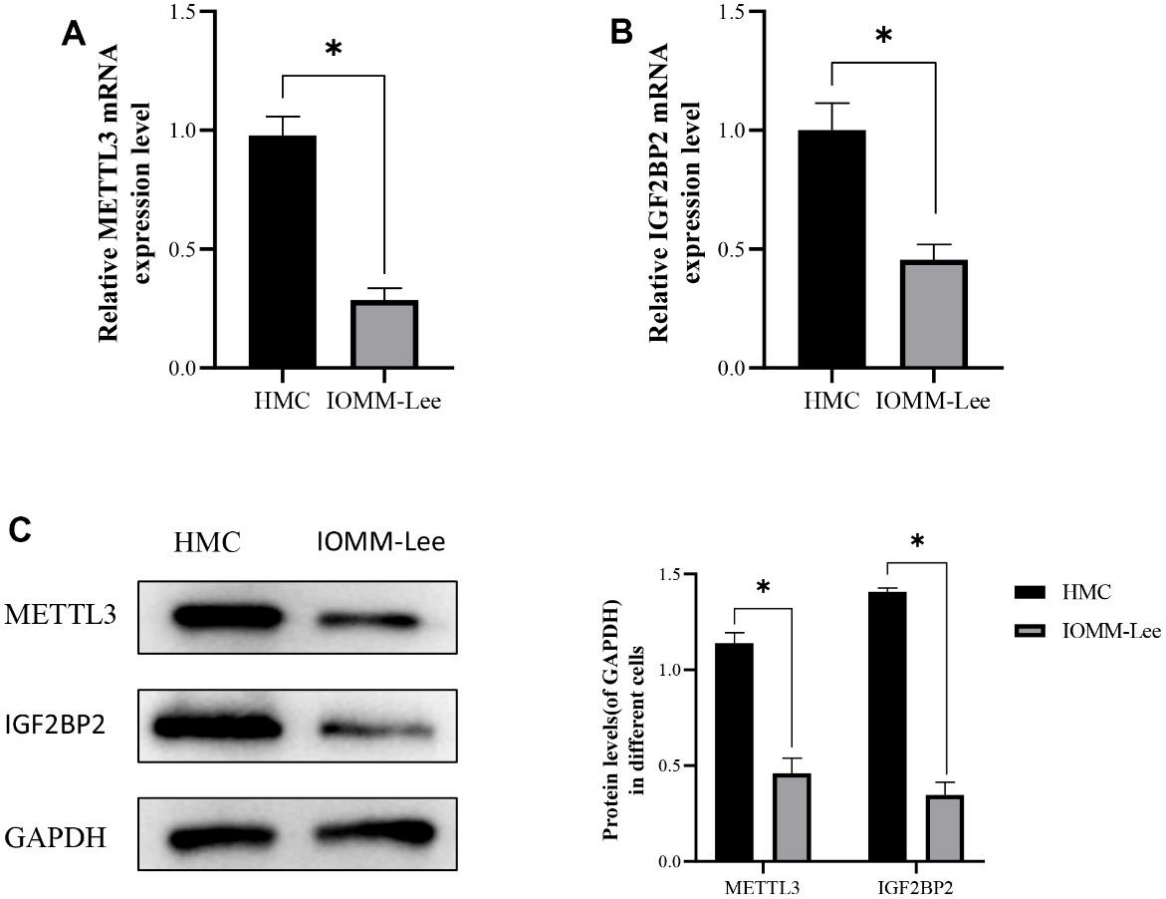
**The expression of METTL3 and IGF2BP2 in meningioma cells.** (**A**) The expression level of METTL3 mRNA in meningioma cells. (**B**) Expression levels of IGF2BP2 mRNA in meningioma cells. (**C**) Protein expression of METTL3 and IGF2BP2 in meningioma cells. *<0.05, **<0.01, ***<0.001.

To verify whether m6A methylated hub regulators (METTL3 and IGF2BP2) played an important role in meningioma, their mRNA expression levels in normal meningeal cells (HMC) and meningioma cells (IOMM-Lee cells) were first detected by reverse transcription polymerase chain reaction (Figure 7A, 7B). The results showed that the expression levels of METTL3 and IGF2BP2 in IOMM-Lee cells were lower than those in HMC. In addition, low expression of METTL3 and IGF2BP2 in meningioma cells was verified by western blotting (Figure 7A).

## DISCUSSION

Meningiomas, as primary intracranial tumors are fatal to patients’ lives. The expression of RNA methylation, an epigenetic regulatory factor, is closely related to the mechanism of tumors and disease progression. In 2017, based on the new classification of DNA copy number analysis, mutation analysis, and RNA sequencing, current understanding of meningioma recurrence and prognosis deepened considerably [11, 12]. In this study, an m6A RNA methylation regulator was used to construct a clinical prognostic model, and factors prominently related to the prognosis of patients with meningiomas were identified. A gene expression landscape featuring m6A RNA methylation regulators was constructed to promote understanding of the mechanism and regulation of methylation in meningiomas. The mRNA and protein levels in HMC and IOMM-Lee cells were verified.

In this study, METTL3 and IGF2BP2 were identified as crucial m6A genes for meningioma prognosis. Previous studies [13, 14] have confirmed that m6A binding proteins (m6A readers) that target RNA were recruited, and methyltransferase complexes (m6A writers) were installed on target RNA through the methyl group of S-adenosylmethionine transferase. Demethylases dynamically modified m6A by removing the methyl group of m6A. Recent study results have further explained the prognostic effects of m6A RNA methylation [15, 16].

The full name of METTL3 is methyltransferase-like protein 3, also known as the N6-adenosine-methyltransferase complex catalytic subunit. The IGF2BP protein (insulin-like growth factor 2 mRNA binding protein) is a unique m6A reader that does not promote mRNA degradation like YTH domain family proteins, but makes mRNA more stable. The results of this study highlight the need for further verification and investigations on the complex biological mechanisms of these m6A targets in meningiomas.

METTL3 catalyzes the modification of m6A. It contains 580 amino acids and consists of a zinc finger domain and a methyltransferase structure domain composition [17]. Chen et al. [18] demonstrated that, in a TTL3 knockout mouse model, METTL3 inhibited the progression of CRC cells by inhibiting m6A-GLUT1-mTORC1. In a study by Liu et al. [19], METTL3 was found to be an independent prognostic factor for relapse-free survival, and the upregulation of METTL3 was closely related to CNV and DNA methylation. IGF2BP2 is also an m6A reader and is closely associated with the prognosis of pancreatic cancer [20, 21]. In addition, lncRNAs have a positive effect on aerobic glycolysis in colorectal cancer, which is achieved by stabilizing IGF2BP2 [22]. These results are consistent with those of the present study. However, modification and regulation of a single m6A gene cannot be consistent for an entire organism. Thus, the prognostic effect of multiple m6A RNA regulators in meningioma was further analyzed, and an m6A methylation regulation network was constructed.

The LASSO results, as well as the univariate and multivariate Cox regression results, confirmed that the RiskScore, was an independent prognostic factor of meningioma and a good indicator of meningioma survival that had a higher significance of sensitivity and specificity. The high- and low-risk groups in the present study, based on methylation regulators, stimulated interest in meningioma pathogenesis. Therefore, the co-expressed gene network related to the RiskScore was further explored, and the entire methylation gene regulation map was investigated.

WGCNA is considered a method for identifying highly correlated genes and characteristics of external samples [23–25]. In this study, the red module was filtered out and negatively correlated with the RiskScore; the 100 genes in the red module were closely related to the RiskScore. The results of GO and KEGG analyses of the genes in the red module also indicated the mechanism by which methylation regulates meningiomas. In general, the results suggested that the characteristic expression of m6A methylated genes is as a prognostic indicator of meningiomas. These m6A genes and their related pathways could serve as potential therapeutic targets for meningiomas. Low expression of METTL3 and IGF2BP2 in meningioma cells (vs. normal meningioma cells) was also confirmed at the transcriptional and translational levels.

However, the present study had some limitations. First, the sample size was insufficient because many cases lacked clinical information. Second, the description of the potential mechanism of these upregulated genes in the prognosis was not based on experimental evidence. Third, confirmation regarding the prognostic importance of METTL3 and IGF2BP2 in clinical patient samples is expected in the future. In conclusion, two m6A methylated genes (METTL3 and IGF2BP2) were identified and a RiskScore methylation regulatory network was constructed, which can be considered prognostic factors for meningioma.

## MATERIALS AND METHODS

### Bioinformatics analysis

The GEO database is a comprehensive and free public gene expression database. Here, three datasets, GSE16581 [26] (clinical information in Table 2), GSE55609, and GSE43290 [27], were included (Supplementary Table 1). Overall, 20 m6A methylation regulators were obtained from previous studies [28, 29], and the expression of GSE16581 was matched separately with the m6A methylation regulators.

**Table 2:** Clinical information of GSE16581 dataset.

**Characteristic**	**Freq**
Age, median (interquartile range)	64(53 to 76)
Gender, No. (%)	
Female	40 (62%)
Male	25 (38%)
RFS_status, No. (%)	
No recurrence	57 (88%)
recurrence	8 (12%)
OS_time, median (interquartile range)	1757(1089 to 2535)
OS_status, No. (%)	
alive	55 (85%)
death	10 (15%)
grade, No. (%)	
Grade I	43 (66%)
Grade II	18 (28%)
Grade III	4 (6%)

### Construction of the prognostic characteristics of methylation regulators

An expression matrix of 20 m6A methylation regulators was obtained from the GSE16581 dataset. First, univariate Cox regression analysis was used to identify m6A methylation regulators related to the prognosis of meningioma (*p* < 0.2 was used as the screening condition). Thereafter, multivariate Cox regression analysis was then used to assess independent prognostic factors associated with overall survival in patients with meningioma (with *p* < 0.05 as a screening condition). The LASSO Cox regression algorithm was used to construct the best prognostic prediction model for the m6A methylation regulators. The optimal cutoff value was defined by the expression level of the m6A methylation regulators (“cutoff” package, version 1.3). Patients with meningioma were divided into high- and low-risk groups based on the RiskScore value. The glmnet installation package in the R environment was used to perform LASSO regression analysis. A prognostic prediction model was constructed based on the m6A methylation regulators. Univariate (*p* < 0.05) and multivariate Cox (*p* < 0.05) regression analysis was used to evaluate the prognostic effect of RiskScore, and Kaplan–Meier curve analysis was performed using the log-rank test. The TimeROC package (version 0.4) was used to compare the predictive accuracy of the RiskScore of genetic characteristics of patients with meningioma, and *p* < 0.05 was considered to indicate statistical significance.

### WGCNA

To better reveal the mechanism of m6A methylation in meningiomas, a gene co-expression network was established using the WGCNA package (version:1.71). This network identified genes closely related to the RiskScore and aimed at constructing a methylation gene regulatory network. First, the optimal cutoff value was determined through the “cutoff” package, and depending on the RiskScore value, high and low expression groups were established. All samples were clustered to identify outliers (height = 100), and PickSoftThreshold was used to determine the soft threshold (β = 4) to construct a scale-free topology model (R^2^ > 0.85). To minimize the influence of noise, the adjacency matrix was converted into a topological overlap matrix (TOM). TOM-based dissimilarity was used to cut the module through a dynamic tree. The number of genes in the smallest module was 30 and cutting height was 0.25. The correlation analysis between the m6A regulator, color module, and genes in the module was visualized. The clusterProfiler installation package (version 4.3.3) was used to perform KEGG pathway enrichment and GO analyses of the genes in the RiskScore module.

### Construction of the m6A gene network in meningioma

The gene in the red module was explored using the online PPI website (STRING); the network was visualized using Cytoscape_v3.8.2 and processed using cytoHubba. Finally, an m6A gene regulation network was constructed for meningiomas.

### RNA extraction and real-time fluorescent quantitative PCR detection

Both meningioma and normal meningioma cells were purchased from the Cell Resource Center of the Shanghai Institute for Biological Sciences, Chinese Academy of Sciences. Total RNA was extracted from meningioma and normal meningioma cells using TRIzol reagent (Invitrogen, USA) according to the manufacturer’s instructions, and complimentary DNA was synthesized from 1 μg of total RNA using the PrimeScript μRT kit with gDNA eraser (Takara, Japan). The expression of METTL3 and IGF2BP2 mRNA was detected by quantitative reverse transcription PCR, using the SYBR premixed dimer eraser, and GAPDH was used as a normalization control. The following primers were used: METTL3 F: 5'-TCTTCAGCATCGGAACCAGC-3’, R: 5'-TGGGGATTTCCTTTGACACCAA-3’. IGF2BP2 F: 5'-GACAGGTCCTGCTGAAGTCC-3’, R: 5'-TGTTGACTTGTTCCACATTCTCC-3’. GAPDH F: 5′-CCCATCACCATTCCAGGAG-3′, R: 5′-GTTGTCATGGATGACCTTGGC-3′.

### Western blot

Protein concentrations were estimated using the bicinchoninic acid protein assay. Total protein was extracted using cold RIPA buffer containing phenylmethanesulfonyl fluoride (Beyotime, China) and phosphatase inhibitor cocktail 2 (Sigma, USA) for 30 min on ice and at 12,000 × *g* at 4° C. The mixture was centrifuged for 20 min. Antibodies against METTL3 (Cat. No. 67733-1-Ig, 1:10000) and anti-IGF2BP2 (Cat. No. 11601-1-AP, 1:8000) were purchased from Proteintech (China). The GAPDH antibody (Cat. No. 11601-1-AP, 1:5000) was used as a normalization control.

### Statistical analysis

R version 4.0.3 software (R Core Team (2020); R: Language and environment for statistical computing; R Foundation for Statistical Computing, Austria: https://www.R-project.org/) was used for data preprocessing, DEG screening, WGCNA, and functional enrichment analysis. One-way ANOVA followed by Dunnett’s multiple comparisons test was performed, using GraphPad Prism software version 8.0.0 for Windows (GraphPad Software, USA; https://www.graphpad.com), to analyze the expression of selected genes in different groups. The “pROC” R package (version 1.18.0) calculated the ROC curve and the AUC to evaluate the diagnostic value of RiskScore.

### Data availability statement

The data used in this study were downloaded from the GEO database (https://www.ncbi.nlm.nih.gov/gds/?term), and the data used to support the results of this study were obtained from the corresponding authors.

## Supplementary Material

Supplementary Tables
