# Supplementary material for: The role of m6A RNA methylation regulator in meningioma
Source: Aging (Albany NY). 2023 Oct 31;15(21):12068–84. doi: 10.18632/aging.205163 (PMC10683626; doi:10.18632/aging.205163)
Supplement: Supplementary Tables [file aging-15-205163-s001.pdf]

SUPPLEMENTARY TABLES

Supplementary Table 1. Basic information of three datasets.

Datasets	Samples	Authors	GPL platform
GSE16581	meningioma:68	Lee Y et al.	GPL570
GSE55609	normal:3, meningioma:21	Yeh TH et al.	GPL570
GSE43290	normal:4, meningioma:47	Tabernero MD et al.	GPL96

Supplementary Table 2. Genes in red module.

Red module
LPHN3
LMO4
SLC38A4
LRRC17
PLA2G4A
HERC5
FYN
SH3BGR
SULT1C4
SLC16A1
RARRES3
GJA1
TNFSF10
BEND6
PIR
PPAP2C
ACSL5
EPB41L2
FBXO15
LINC00893
CXCL11
SNCAIP
IMPA2
NINJ2
SYNC
PC
AMPH
BEND5
EFCAB7
ADAMTSL1
CCDC132
CDC14A
PRMT6
ARNTL
MAB21L2
TBC1D32
IFNGR1
PGM3
ZMYND12
CNN3
NMI
TPR
COX4I2
MIER1
PPIL4
SBF2-AS1
AKR7A2
FGGY
POLI
CD58

ZBED5-AS1
FAIM
LINC00959
SCO2
HSF2
CDC20
NT5DC1
IFI35
PEX3
THNSL1
CCDC28A
CHM
WTAP
EXOC4
TAF12
FAF1
VAMP3
ANKRD13C
SNX30
MED30
UBE4B
COL14A1
C6orf57
PROSC
WRAP73
DDX6
RBM26-AS1
DPY19L1
CYB5D2
NDRG3
ZUFSP
DENND2C
NEU1
ADPRHL2
ANXA7
MAPRE2
CAMTA1
OSBPL9
HSPB11
ZNF593
EFHC1
C6orf203
LOC102606465
ZSWIM7
RHOC
RP11-676J12.6
DUSP23
PINK1
STX7
FIG4
